# The accuracy of the auto-stop function of different endodontic devices in detecting the apical constriction

**DOI:** 10.1186/s12903-017-0425-y

**Published:** 2017-11-29

**Authors:** David W. Christofzik, Andreas Bartols, Mahmoud Khaled, Birte Größner-Schreiber, Christof E. Dörfer

**Affiliations:** 1 School for Dental Medicine, Christian-Albrechts-University Kiel, Clinic for Conservative Dentistry and Periodontology, Arnold-Heller Str. 3, 24105 Kiel, DE Germany; 2 Dental Academy for Continuing Professional Development, Lorenzstraße 7, 76134 Karlsruhe, DE Germany

**Keywords:** Apex locator, Apical constriction, Raypex 6, Reciproc, EndoPilot

## Abstract

**Background:**

Electronic apex locators (EALs) are modern devices used to determine the working length during root canal preparation. The newest endodontic motors provide an integrated EAL with auto-stop function to prevent instrumentation beyond the predefined working length during rotary root canal preparation. The aim of this study was to compare the accuracy of the auto-stop function of the VDW.Gold RECIPROC motor (VDW, Munich, Germany), the EndoPilot motor (Schlumbohm, Brokstedt, Germany) and the manual measurement with Raypex 6 (VDW, Munich, Germany) to detect the apical constriction (AC).

**Methods:**

Ninety human teeth were chosen and randomly assigned to three experimental groups (30 teeth each): VDW.Gold RECIPROC motor continuous measuring (RCM), EndoPilot continuous measuring (ECM) and Raypex 6 manual measuring (RMM). When the measurement file reached the AC, the file was fixed in the tooth. The tooth was embedded in acrylic resin and the root tip was exposed, so that the histologic structure of the root canal and the file tip was visible for microscopic analysis. Afterwards, the distance of the file tip to the AC (D_AC_) was automatically computed with a specially developed software tool.

**Results:**

The mean D_AC_ were −13.18 μm (SD 88.46 μm) for RMM, −22.70 μm (SD 91.57 μm) for RCM and 18.74 μm (SD 88.11 μm) for ECM. The differences were not statistically significant (*P* = 0.181). The rates for instrumentation beyond the AC were not statistically different (Chi^2^ = 4.753, *p* = 0.096).

**Conclusions:**

All measurement methods showed a high accuracy in detecting the AC. The auto-stop function of these endodontic motors is a reliable addition to the endodontic armamentarium.

## Background

Loss of working length (WL) during preparation of root canals can lead to instrumentation beyond the predefined WL [1–3]. This effect is mainly attributed to straightening of the root canal during instrumentation. Some studies showed, that this kind of over instrumentation as well as under instrumentation can negatively affect the outcome of the endodontic treatment [4–6], while it remains unclear what exactly is the most favourable extent of the apical limit of root canal preparation [7, 8]. Moreover, all endodontic instruments produce apically extruded debris [9–11], even when the preparation stays within the confines of the root canal. Therefore, preparations ending in the periapical tissue will lead to a greater amount of extruded debris that could trigger a neurogenic inflammatory response resulting from an irritation of the periodontal ligament [12] with following postoperative symptomatic apical periodontitis. For this reason, it seems favourable to control the determined WL during root canal preparation to avoid preparations ending in the periapical tissue.

The most commonly practiced electronic determination of endodontic WL is done manually. Usually a small sized stainless steel hand instrument, e.g. an ISO 10 or 15 K-file, is connected to an electronic apex locator (EAL) and introduced into the root canal. When the WL is determined, this length is transferred to the subsequent instruments, e.g. rotary nickel-titanium (NiTi) instruments. Normally WL must be repeatedly controlled manually from time to time to prevent instrumentation beyond the WL [7]. Endodontic motors with integrated apex locators offer a solution to the described problem. These motors continuously measure the WL during canal preparation and contain an auto stop function when WL is reached.

It has been demonstrated in many studies that WL determination with apex locators is generally reliable [13–16]. Some older endodontic devices with continuous WL control have been evaluated earlier [17–20]. The TriAuto ZX (J Morita, Tokyo, Japan) endodontic hand piece as well as the Root ZX II (J Morita, Tokyo, Japan) showed acceptable results in determining the WL during rotary root canal preparation [17, 18]. Recently, a study evaluated the accuracy of the newer VDW.Gold RECIPROC motor (VDW, Munich, Germany) WL determination during reciprocating motion and found no differences to other apex locators [21]. The EndoPilot (Schlumbohm, Brokstedt, Germany) is another new generation endodontic motor with continuous WL measuring function. The implemented apex locator uses a new detection method based on an impulse measuring system that should allow a greater accuracy to detect the apical constriction (AC), especially in the presence of sodium hypochlorite (NaOCl) in the root canal [22].

The aim of this study was to compare the accuracy to detect the AC during rotary root canal preparation with the VDW.Gold RECIPROC motor, the EndoPilot and the manual detection of the AC with Raypex 6 (VDW, Munich, Germany) and the following transfer of the measured length to rotary preparation. Our null hypothesis was: The detection of the AC with the three methods shows no differences.

## Methods

### Samples

Ninety caries-free, unrestored permanent adult teeth with completely formed roots, which were extracted for surgical or periodontal reasons without the option of conservation, were chosen for this study. All teeth were collected with written consent of the patients and under an ethic protocol approved by the IRB of the Christian-Albrechts-University Kiel (Ethics committee of the Christian-Albrechts-University Kiel D444/10).

The teeth were block-randomized to three experimental groups. In every group 10 single rooted, 10 double rooted and 10 molar teeth were used. In multi-rooted teeth only the mesiobuccal or mesiolingual root canal was used. Therefore in every group the shares of straight and curved and single and multiple rooted teeth were equal. The teeth were allocated to the three groups (each *N* = 30): VDW.Gold RECIPROC motor continuous measuring (RCM), EndoPilot continuous measuring (ECM) and Raypex 6 manual measuring (RMM).

In all teeth the primary access cavity was prepared with diamond-coated burs (Brasseler, Lemgo, Germany) and an occlusal plateau was prepared as a clear reference. All root canal orifices were initially preflared with Gates Glidden burs #2 and #3 (Brasseler, Lemgo, Germany). The pulp chambers were irrigated with 3% NaOCl. Then the root canals were scouted with ISO 06 and 08 K-Files (VDW, Munich, Germany) down to the apical third.

### Electronic measurements of the working length

**Fig. 1:**
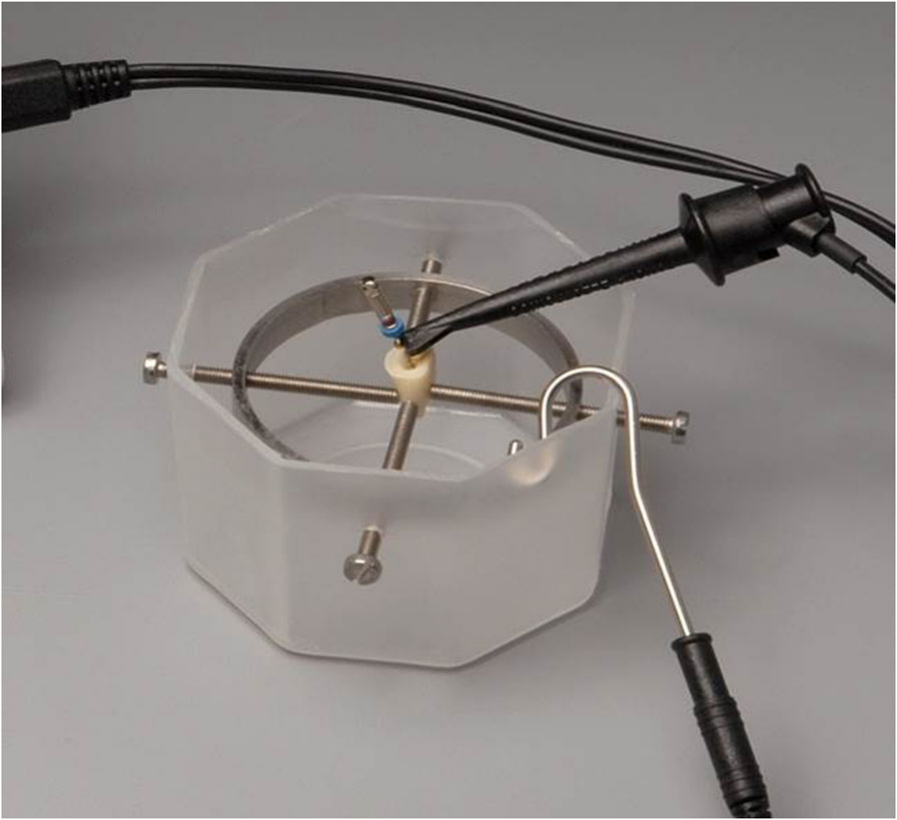
Experimental setting with tooth fixed for detecting the apical constriction. The roots were covered with 0.9% NaCl solution

An established model was used to perform the working length (WL) measurements [14]. The teeth were fixed in a measurement device that was filled with 0.9% NaCl-solution. The roots were completely covered by the solution (Fig. 1).

In the Raypex 6 group (RMM) an ISO 10 K-hand file (VDW, Munich, Germany) was used for manual detection of the apical constriction (AC). According to the manufacturer’s instructions, this is the case when the display screen indicates the third green line. The length to AC was marked with the rubber stopper at the ISO 10 K-file and measured with an endodontic Minifix ruler (VDW, Munich, Germany) and rounded off to a precision of 0.25 mm to avoid instrumentation beyond WL. Then the measured length was transferred with the same ruler to a #10/04 Mtwo-file (VDW, Munich, Germany). This file was used in rotary motion to the transferred length. So we mimicked the generally established clinical protocol for WL determination.

In the VDW.Gold RECIPROC (RCM) and Endopilot (ECM) groups, #10/04 Mtwo-files (VDW, Munich, Germany) were used in rotary motion until the auto-stop function of these motors indicated that the AC was reached. This is the case when the third green LED on the VDW. Gold RECIPROC motor lights and accordingly for the EndoPilot when the apex line is reached and the apex value is 36 on the display screen of the device.

When the AC was reached, the files were fixed in the tooth with a flowable composite (Tetric EvoFlow, Ivoclar Vivadent, Ellwangen, Germany). Afterwards, the teeth were embedded in acrylic resin (Technovit 4071, Heraeus Kulzer, Wehrheim, Germany) to fix them for further processing. The acrylic blocks with the embedded teeth were ground (ATM Saphir 360 E, ATM, Mammelzen, Germany) under microscopic control (STEMI, Zeiss, Oberkochen, Germany) until the maximum width of the root canal was visible. The apical region with the tip of the measurement file was photographed with a digital camera (CFW 1312 M, Scion, Frederick, USA) mounted to a microscope (Axiophot 2, Zeiss, Jena, Germany). The images were adjusted and scaled with AxioVision (Zeiss, Jena, Germany).

### Measurements of the apical region

**Fig. 2:**
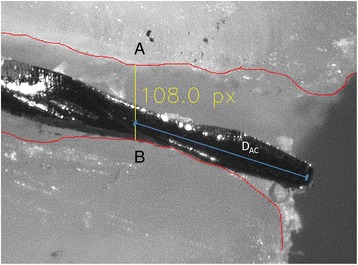
Detection of the apical constriction (AC) (distance **a** – **b**) and measurement from instrument tip to AC

All adjusted images were further edited with Adobe Photoshop CS 5.1 (Adobe, Munich, Germany). The root canal walls were traced with a red coloured line (RGB 255,0,0). An experimental software detected the smallest distance between the two root canal walls (Fig. 2) and measured the diameter of the AC. Now it was possible to measure the distance of the instrument tip to the intersection with the AC. For these metrical analyses the correct μm per pixel relation was used to calculate the distances in μm. The exact analytical procedure was already described in an earlier publication [23].

### Statistical analysis

The statistical analysis was performed with GraphPadPrism 6 (GraphPad Software, Inc. La Jolla, CA, USA). Analysis of variance (ANOVA) was used to compare the different groups concerning mean D_AC_. Pearson-Chi-Square test was used to compare the groups for the frequencies of instrumentation beyond WL. The α-type error was set to 0.05.

## Results

In the RMM group 28 teeth, in the RCM group 29 and in the ECM group 30 teeth were included for statistical analysis. Three teeth were excluded, because the apical foramen was too large and did not allow a reproducible measurement.

The mean distances D_AC_ were −13.18 μm (SD 88.46 μm) for RMM, −22.70 μm (SD 91.57 μm) for RCM and 18.74 μm (SD 88.11 μm) for ECM. The differences were not statistically significant (*P* = 0.181) (Table 1). Negative values indicate a position of the root canal instrument beyond (apical to) the AC and positive values indicate a position above (coronal to) the AC. There were no statistically significant differences in the frequencies of preparation beyond WL (Chi^2^ = 4.753, *P* = 0.096).

**Table 1:** Mean distance of file tip from the apical constriction (D_AC_) in μm and frequency of instrumentation beyond the apex (FbA)

	RCM	ECM	RMM
D_AC_ (SD)	−22.70 (91.57)^a^	18.74 (88.11)^a^	−13.18 (88.46)^a^
FbA (%)	17 (59)^a^	12 (40)^a^	19 (68)^a^
N	29	30	28

Values with the same superscript letter (a) in rows were not statistically different (*P* > 0.05)

ANOVA for D_AC_ and Pearson-Chi-Square for FbA

We also conducted a statistical analysis of the diameters of the ACs in all three test groups. The mean diameters were in the RCM group 43.64 μm (SD 30.61 μm), in the ECM group 41.46 μm (SD 26.72 μm) and in the RMM group 46.01 μm (SD 23.25 μm). There were no significant differences between the groups (Kruskal-Wallis test with Dunn’s multiple comparisons test all *p* > 0.94) showing an equal distribution of the diameters of the ACs.

## Discussion

The manual technique with RMM as well as the auto-stop methods of RCM and ECM with rotary instruments detected the AC very precisely. There were no statistical differences between the different experimental groups in the mean aberrations from the AC. Therefore our null hypothesis was not rejected.

A lot of studies evaluate the electronically determined WL as the aberration within 0.5 mm to a predefined target area [24–27], as this is a unit that can be measured clinically. In this study, the mean values of aberration from the AC were within the range of about ±10 to 20 μm and even the maximum aberrations in all test groups were well within the range of ±0.5 mm. One review reported mean aberrations of 0.25 mm (SD 0.17 mm) to 1.36 mm (SD 0.41 mm) from the instrument tip to the AC for manual measurements [8]. But the reviewed measurement devices were of older generations. One study compared the accuracy of the integrated apex locator of the RCM in reciprocating mode with two other manual apex locators [21]. There were no statistical differences between different measurement techniques, which is in concordance with our results. However, the target area was not the AC but a root canal with a standardized length. So far there is only one study of our research group that assessed the EAL function of the EndoPilot device [23]. Mean distances to the AC were not different for manual Raypex and EndoPilot measurements as well as for EndoPilot in rotary mode. But EndoPilot in rotary mode made significantly more preparations beyond WL than the other methods. In this study, ECM showed less tendency towards such over preparation than RCM and RMM, but the overall frequencies of preparation beyond WL were not statistically different.

Earlier generations of endodontic handpieces or motors with integrated EALs and auto-stop function have already shown acceptable results in different studies [17, 18, 25, 28]. Two studies found that the Tri Auto ZX endodontic handpiece (Morita, Kyoto, Japan) when used in the auto reverse mode with certain settings, lead to enlargement of the AC in a high frequency [17, 18]. We also detected a tendency to preparation beyond WL in continuous measuring mode with RCM (59%) and less pronounced with ECM (40%). Moreover, with ECM the mean D_AC_ had a positive value, indicating a position of the instrument tip coronal to the AC. Perhaps this can be explained by the higher frequency of measurements (that is about 50 times higher) compared to the other devices, so that the auto-stop function is being triggered faster. However, all preparations beyond WL were within a limit of 250 μm apical of the AC.

Interestingly, also the transfer of the manually determined root canal length with RMM to the rotary file and the following rotary preparation without auto-stop function did not lead to a higher inaccuracy in detecting the AC than the preparation techniques with auto-stop function. As the transfer of the measurements was done under simulated clinical conditions with only a precision of 0.25 mm of the endodontic ruler, a higher rate of inaccurate preparations was expected. To the authors’ best knowledge, there are no studies so far that investigated the transfer error from manual WL determination to rotary instrumentation.

A weakness of our study may be the in vitro study design. The clinical situation can alter the function of apex locators because of patient related factors like tooth morphology, metallic restorations, bleeding, suppuration of the root canal or even the irrigant used [28]. But models for evaluation of electronic apex locators are described widely in literature [14, 18, 29] and simulated the clinical situation sufficiently [30]. Moreover, the use of different types of roots may raise some concerns if the experimental conditions for the different test groups were homogenous enough. But it should be considered that under clinical conditions there is no possibility to assess the exact type of the measured AC. Therefore, the operator has to rely on the measurement of the apex locator regardless of the type of root. In this experimental model such different situations were simulated. The homogeneity of the groups was secured by block randomization of the different root types. Additionally, we observed a mean precision in our measurements being more accurate than the precision of previously published studies [8].

The strength of this study is the precision of our specially developed measurement method. It allows the detection of the tip of the measurement instrument in relation to the AC in terms of μm instead of mm. The method has also a high reproducibility [23] because of the automatically computed measurements and is therefore not prone to subjectivity, but does not necessarily reflect the imprecision of the clinical situation.

## Conclusions

The manual detection of the AC with RMM as well as the detection of the AC during continuous measurement of WL in rotary mode with RCM and ECM resulted in a high degree of accuracy with only small aberrations from the targeted area. Therefore, the auto-stop function of the tested devices is a valuable addition to the endodontic armamentarium and enhances the safety of rotary root canal preparation.

## Abbreviations

AC: Apical constriction
D_AC_: Distance from apical constriction
EAL: Electronic apex locator
ECM: Experimental group: EndoPilot motor in continuous measuring mode
FbA: Frequency of instrumentation beyond the apex
NaCl: Sodium chloride
NaOCl: Sodium-hypochlorite
NiTi: Nickel-titanium
RCM: Experimental group: Reciproc motor in continuous measuring mode
RMM: Experimental group: Raypex 6 manual measuring mode
WL: Working length
